# Chromogranin A‐positive hormone‐negative endocrine cells in pancreas in human pregnancy

**DOI:** 10.1002/edm2.223

**Published:** 2021-01-06

**Authors:** Abu Saleh Md Moin, Kylie Zeng, Robert A. Rizza, Sangeeta Dhawan, Alexandra E. Butler

**Affiliations:** ^1^ Diabetes Research Center (DRC) Qatar Biomedical Research Institute (QBRI) Hamad Bin Khalifa University (HBKU) Qatar Foundation (QF) Doha Qatar; ^2^ Larry L. Hillblom Islet Research Center David Geffen School of Medicine University of California Los Angeles Los Angeles CA USA; ^3^ Division of Endocrinology Diabetes, Metabolism, and Nutrition Mayo Clinic College of Medicine Rochester MN USA; ^4^ Diabetes and Metabolism Research Institute City of Hope Duarte CA USA

**Keywords:** beta cell, pancreas, pregnancy, regeneration

## Abstract

**Introduction:**

We sought to determine whether chromogranin A‐positive hormone‐negative (CPHN) endocrine cells are increased in the pancreas of pregnant women, offering potential evidence in support of neogenesis.

**Methods:**

Autopsy pancreata from pregnant women (*n* = 14) and age‐matched non‐pregnant control women (*n* = 9) were obtained. Staining of pancreatic sections for chromogranin A, insulin and a cocktail of glucagon, somatostatin, pancreatic polypeptide and ghrelin was undertaken, with subsequent evaluation for CPHN cell frequency.

**Results:**

The frequency of clustered β‐cells was increased in pregnant compared to non‐pregnant subjects (46.6 ± 5.0 vs. 31.8 ± 5.0% clustered β‐cells of total clustered endocrine cells, pregnant vs. non‐pregnant, *p* < .05). Frequency of endocrine cocktail cells was lower in pregnant women than non‐pregnant women (36.2 ± 4.0 vs. 57.0 ± 6.8% clustered endocrine cocktail cells of total clustered endocrine cells, pregnant vs. non‐pregnant, *p* < .01). No difference in frequency of CPHN cells was found in islets, nor in clustered or single cells scattered throughout the exocrine pancreas, between pregnant and non‐pregnant women. The frequency of CPHN cells in pregnancy was independent of the number of pregnancies (gravidity).

**Conclusions:**

Our findings of no increase in CPHN cell frequency in pancreas of pregnant women suggest that this potential β‐cell regenerative mechanism is not that by which the increased β‐cell mass of pregnancy is achieved. However, an increase in the percentage of clustered β‐cells was found in pregnancy, with decreased frequency of other endocrine cells in clusters, suggesting a compensatory shift from other pancreatic endocrine cell types to β‐cells as a mechanism to meet the increased insulin demands of pregnancy.

## Graphical abstract

Chromogranin A‐positive hormone‐negative (CPHN) endocrine cells are increased in the pancreas of pregnant women, offering potential evidence in support of neogenesis; we found no increase in CPHN cell frequency in pancreas of pregnant women suggesting that this potential β‐cell regenerative mechanism is not that by which the increased β‐cell mass of pregnancy is achieved. However, an increase in the percentage of clustered β‐cells was found in pregnancy, with decreased frequency of other endocrine cells in clusters, suggesting a compensatory shift from other pancreatic endocrine cell types to β‐cells as a mechanism to meet the increased insulin demands of pregnancy.

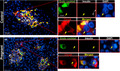

## 1 INTRODUCTION

We have previously reported a novel endocrine cell type in the pancreas that expresses chromogranin A but none of the other known pancreatic hormones. We adopted the term chromogranin A‐positive hormone‐negative (CPHN) cells and found that they were increased in diabetes and other pancreatic disorders.^1^, ^2^, ^3^, ^4^, ^5^ The distribution of CPHN cells, occurring mainly as single cells or in small clustered groups of 1–3 cells scattered throughout the exocrine pancreas, rather than in established islets, appeared to recapitulate what is found in the pancreas during early human development^1^ and led us to conclude that these were newly forming cells.

However, there is an alternative explanation: that CPHN cells are endocrine cells demonstrating an altered identity that occur with increased frequency in the setting of hyperglycaemia in type 1 and type 2 diabetes. It is known that pancreatic endocrine cells can undergo various ‘cell plasticity’ events in response to physiological stresses.^6^ There is also growing evidence that pancreatic endocrine cells may alter their identity (either by dedifferentiation or transdifferentiation) in response to metabolic abnormalities^7^, ^8^ with alpha to beta transdifferentiation having been described, albeit mainly in mice.^9^

Mammalian pregnancy is marked by a stepwise adaptation of the endocrine pancreas with expansion of maternal insulin secretory capacity as the primary adaptation that is necessary to allow the foetus preferential use of circulating glucose.^10^, ^11^, ^12^ The physiological stimulus of pregnancy gives rise to a period of metabolic plasticity, decreasing maternal insulin sensitivity by approximately 50%.^13^, ^14^ To maintain euglycaemia, that insulin resistance is offset by an ~250% increase in maternal insulin production.^13^, ^14^ These physiological adaptations are accomplished partly through increased workload (insulin synthesis and secretory burden) of the existing β‐cells. However, there is also an increase in β‐cell numbers in pregnancy.^12^

The exact mechanism of adaptive expansion of β‐cells in human pregnancy is still under investigation. However, studies in mouse models^15^, ^16^, ^17^, ^18^, ^19^ and in vitro studies with human islets^20^ have revealed that the morphological changes such as replication and increase of pancreatic β‐cell numbers in pregnancy are driven mainly by placental lactogen (PL) and prolactin‐mediated gene regulation.

Chromogranin A‐positive hormone‐negative cells may represent newly forming endocrine cells or pancreatic endocrine cells with an altered identity, perhaps dedifferentiating/transdifferentiating towards a β‐cell fate in response to physiological stress conditions. We therefore questioned whether, in pregnancy, a physiological state of β‐cell stress without the confounding variable of hyperglycaemia, there would be an increase in the frequency of CPHN cells.

To address this question, we analysed sections of pancreata from human autopsy subjects (14 pregnant and 9 non‐pregnant age‐matched women) and sought to determine CPHN cell frequency in the pregnant vs. control women.

## 2 MATERIALS AND METHODS

### 2.1 Autopsy cases

Human autopsy pancreas from 14 pregnant women and 9 age and pre‐pregnancy BMI matched non‐pregnant control women was obtained Table 1, Figure S1A, B). Both the pregnant and the non‐pregnant women have been included in a previous publication.^21^ Subjects were identified in the Mayo Clinic autopsy database (RAR). To be included in the study, each subject had to fulfil the following criteria: complete autopsy within 24 h of death, with pancreas of adequate size and quality collected. If the pancreas tissue quality was suboptimal, due to autolysis or pancreatitis, this was an exclusion criterion. Women included in the study did not have a history of diabetes. Subject demographic data and cause of death are listed in Table 1. Institutional Review Board (IRB) approval from the Mayo Clinic and University of California Los Angeles (UCLA) was obtained. Both the Mayo Clinic IRB and the UCLA IRB waived the requirement for written informed consent for this study due to the following reasons: (1) According to the FDA definition of human subjects, a human subject means a living individual and therefore (2) research on human autopsy subjects is considered by the IRB to be ‘exempt’ as defined by federal regulation 45 CFR 46, as autopsy subjects do not meet the FDA definition of human subjects.

**TABLE 1:** Clinical characteristics of pregnant and non‐pregnant women

Pregnant	Age	Sex	BMI	Cause of death	Weeks of gestation	Foetal viability	Pregnancy history
1	22	F	22.2	Increased intracranial pressure due to encephalitis	22	Not viable	G1P0
2	23	F	24	Thrombotic mitral stenosis	36	Live	G1P1
3	19	F	21.3	Ruptured AV malformation	14	Not viable	G1P0
4	18	F	21.2	Diffuse cerebral oedema (hypoxic encephalopathy)	37	Live	G1P0
5	19	F	23.9	Trauma	15	Stillborn	G2P1
6	36	F	34.1	Myocardial failure secondary to ARDS	27	Stillborn	G2P1
7	25	F	22.5	Trauma	18	Stillborn	x
8	26	F	18.3	Temporal lobe abscess	34	Live	G4P3
9	42	F	28	Phaeochromocytoma, hypertensive CVD	36	Stillborn	G14P13
10	42	F	23.4	IPH, shock and coma	40	Live	G1P1
11	32	F	28	Acute respiratory paralysis secondary to polio	16	Not viable	G5P4
12	21	F	27.8	Overdose	16	Not viable	x
13	31	F	19.9	Sudden death, unexplained	10	Not viable	G3P0
14	25	F	22.2	Acute liver failure (fulminant HBsAg+hepatitis)	22	Not viable	G2P1
Mean	27.2		24.1		24.5		
SEM	2.2		1.1		2.7		

Non‐pregnant	Age	Sex	BMI	Cause of death			
1	19	F	22.6	MVA			
2	22	F	22.9	MVA			
3	18	F	17.7	Airplane crash, spinal trauma			
4	27	F	17	Ruptured saccular aneurysm, respiratory arrest			
5	29	F	18.7	MVA			
6	26	F	20.9	MVA			
7	25	F	23.7	Acute liver failure (hepatitis)			
8	32	F	19.7	Hypertensive pulmonary vascular disease			
9	42	F	25.5	Bronchopneumonia			
Mean	26.7		21.0				
SEM	2.4		1.0				

Abbreviations: ARDS, acute respiratory distress syndrome; AV, arterial venous; CVD, cardiovascular disease; G, gravida; HBsAg, hepatitis B surface antigen; IPH, intrapartum haemorrhage; MVA, motor vehicle accident; P, para.

Fasting blood glucose values were not available in the medical record; therefore, non‐diabetic status was based upon the lack of a history of diabetes.

### 2.2 Pancreatic tissue processing

Autopsies were undertaken at Mayo Clinic. Pancreas tail was sampled (~2.0 × 1.0 × 0.5 cm size) and fixed in formaldehyde together with a sample of spleen before being embedded in paraffin. 4 μm sections were cut from the tissue blocks.

### 2.3 Immunofluorescence staining and quantification of chromogranin A‐positive hormone‐negative [CPHN] cells

Sections were stained for Chromogranin A, insulin, glucagon, somatostatin, pancreatic polypeptide and ghrelin to detect CPHN cells as described previously.^1^, ^3^ In brief, slides were incubated overnight at 4°C with a cocktail of primary antibodies prepared in antibody solution (3% bovine serum albumin in Tris‐buffered saline with 0.2% Tween 20). The primary antibodies were detected by a cocktail of appropriate secondary antibodies (Jackson ImmunoResearch, Westgrove, PA). The information for both primary and secondary antibodies is detailed in Table S1. Mounting medium containing DAPI (Vectashield,Vector Laboratories, Burlingame, CA) was used for counterstaining the slides to mark the nuclei and slides were viewed using a Leica DM6000 microscope (Leica Microsystems, Deerfield, IL). All images were acquired using the ×20 objective (×200 magnification) and using a Hamamatsu Orca‐ER camera (catalog no. C4742‐ 80‐12AG,Indigo Scientific, Bridgewater, NJ) and Openlab software (Improvision, Lexington, MA).

For determination of pancreatic endocrine cells in the non‐pregnant and pregnant subjects, 50 islets (an islet was defined as a grouping of four or more endocrine cells), all clustered (a cluster was defined as a grouping of three or fewer chromogranin positive cells) and single endocrine cells contained within the designated areas were imaged at 20× magnification, counted and recorded. Each field of view was determined to be 0.292 mm^2^. Analysis and quantification of endocrine cells and CPHN cells was performed as in our previous reports.^1^, ^3^ Analysis was performed in a blinded manner by two of the authors (ASMM and KZ) independently,all chromogranin‐positive hormone‐negative (CPHN) cells were confirmed independently by two of the other authors (AEB and SD). The mean number of endocrine cells counted within islets for the pregnant and non‐pregnant women was 2,198 ± 202 and 2,511 ± 231 cells per individual, respectively. The mean number of endocrine cells in clusters in the pregnant and non‐pregnant women was 70 ± 9 and 85 ± 17 cells per individual. The mean number of chromogranin A‐positive hormone‐negative [CPHN] cells identified in islets from pregnant subjects was 34 ± 12 cells per individual and from the non‐pregnant women was 19 ± 11 cells per individual. The mean number of CPHN cells per individual counted in clusters for the pregnant women was 14 ± 6 per individual and for the non‐pregnant women was 8 ± 2 cells per individual.

To calculate the % of total clustered endocrine cells, the denominator used was the total number of endocrine cells counted in all compartments [islet endocrine cells and endocrine cells counted as clustered and as single cells] (total number of clustered endocrine cells in the pancreas sections/total number of endocrine cells in all compartments) X 100. To calculate the % of clustered β‐cells or endocrine cocktail cells, the denominator used was the total number of clustered endocrine cells (number of clustered β‐cells or endo‐cocktail cells/total number of clustered endocrine cells) X 100.

To calculate the % of CPHN cells in islets, the denominator used was the total number of endocrine cells counted in islets (Number of CPHN cells in total 50 islets per subject/Number of total endocrine cells in 50 islets per subject) X 100.

### 2.4 Statistical analysis

Data are presented as means ±SEM. Statistical analysis was performed using GraphPad Prism 8 (GraphPad Software, San Diego, CA, USA).

## 3 RESULTS

### 3.1 The composition of endocrine cells (clustered endocrine cells and endocrine cells in islets) in pregnant vs. non‐pregnant women (Table S2)

**FIGURE 1:**
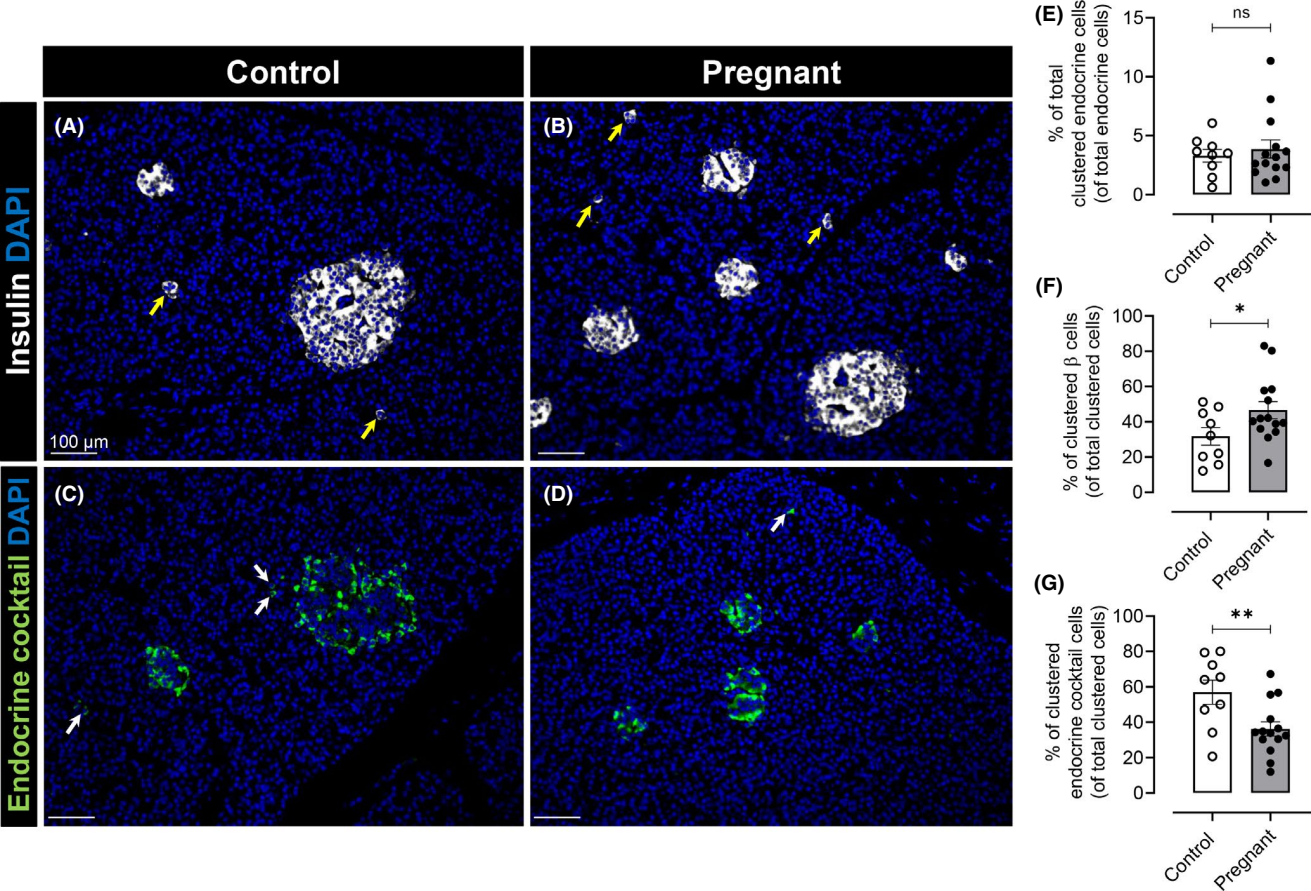
Examples of immunofluorescent images of insulin (A, B) and endocrine cocktail (C, D) immunoreactivity in islets or as clustered cells in four different pancreas sections of non‐pregnant and pregnant cases. Merged images showing insulin in white (A and B) and endocrine cocktail in green (C and D) with DAPI in blue (all images). Yellow arrows indicate clustered β‐cells and white arrows indicate clustered endocrine cocktail cells in both non‐pregnant and pregnant cases. There was no change in frequency of total clustered endocrine cells (of total endocrine cells counted in all compartments of pancreas) in pregnancy (3.8 ± 0.7 vs. 3.5 ± 0.5% of clustered endocrine cells, Pregnant vs. non‐pregnant, p = ns) (E). Frequency of clustered β‐cells (of total clustered endocrine cells) was higher in pregnant cases compared to non‐pregnant cases (46.6 ± 5.0 vs. 31.8 ± 5.0% of clustered β‐cells, pregnant vs. non‐pregnant, *p* < .05) (F). The frequency of clustered endocrine cocktail cells (of total clustered endocrine cells) was lower in pregnant cases compared to non‐pregnant cases (57.02 ± 6.8 vs. 36.2 ± 4.02% of clustered endocrine cocktail cells, pregnant vs. non‐pregnant, *p* < .01) (G). Scale bar, 100 μm (all images)

Consistent with our previous report,^21^ islet density with increased number of smaller islets with clustered β‐cells was observed in pancreas of pregnant women compared with non‐pregnant women Figure 1A,B, Table S2. In the current study, clustered endocrine cocktail cells were also observed both in control and pregnant cases Figure 1C,D. There was no change in % of total clustered endocrine cells in pancreas in pregnant women compared to non‐pregnant (3.8 ± 0.7 vs. 3.5 ± 0.5% of clustered endocrine cells of total endocrine cells, pregnant vs. non‐pregnant, p = ns) Figure 1E. However, the % of clustered β‐cells was increased in pregnant women compared to non‐pregnant (46.6 ± 5.0 vs. 31.8 ± 5.0% of clustered β‐cells of total clustered endocrine cells, pregnant vs. non‐pregnant, *p* < .05) Figure 1F. The % of endocrine cocktail cells was lower in pregnant women than in non‐pregnant women (36.2 ± 4.0 vs. 57.02 ± 6.8% of clustered endo‐cocktail cells of total clustered endocrine cells, pregnant vs. non‐pregnant, *p* < .01) Figure 1G. No correlation was found between length of gestation and the frequency of clustered beta cells or endocrine cocktail cells in the pregnant cohort.

There was no change in islet composition in pregnant women with regard to the total number of endocrine cells per islet cross section vs. control subjects (42.3 ± 4.1 vs. 50.1 ± 4.4 total endocrine cells/islet section, pregnant vs. non‐pregnant, p = ns Figure S2A). There was also no difference in the number of beta cells per islet cross section in the pregnant vs. non‐pregnant subjects (26.8 ± 2.2 vs. 28.1 ± 2.7 beta cells/islet cross section, pregnant vs. non‐pregnant, p = ns). Figure S2B) or in the number of endocrine cocktail cells (cells that express any of the known pancreatic hormones other than insulin) per islet section (14.8 ± 2.2 vs. 21.6 ± 3.1 endocrine cocktail cells/islet cross section, pregnant vs. non‐pregnant, p = ns) Figure S2C).

### 3.2 The comparison of CPHN cells in islets and clustered cells in pregnant vs. non‐pregnant women Table S3

**FIGURE 2:**
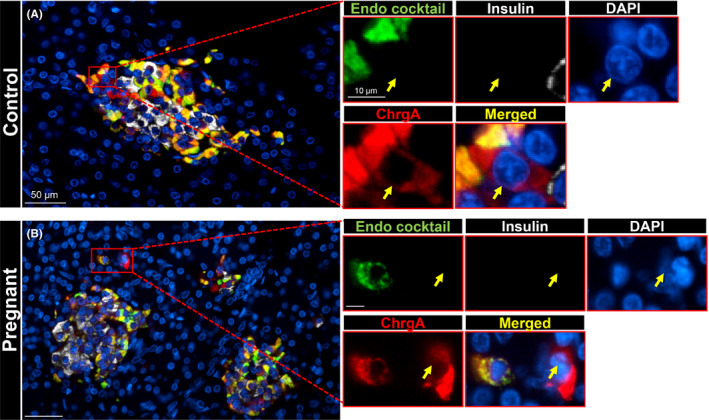
Example of a chromogranin A‐positive hormone‐negative (CPHN) cell in pancreas from a control (non‐pregnant subject) (A) and pregnant subject (B). Individual layers are stained for endocrine cocktail (Glucagon, Somatostatin, Pancreatic Polypeptide and Ghrelin) [green], insulin [white], Chromogranin A (ChrgA) [red] and DAPI [blue] are shown along with the merged image. Insets, magnified images of the desired areas (indicated by red squares) of low power images to clearly demonstrate the CPHN cells. Yellow arrows indicate CPHN cells in both control and pregnant subjects. Scale bars, 50 μm (for low power images) and 10 μm for the insets

**FIGURE 3:**
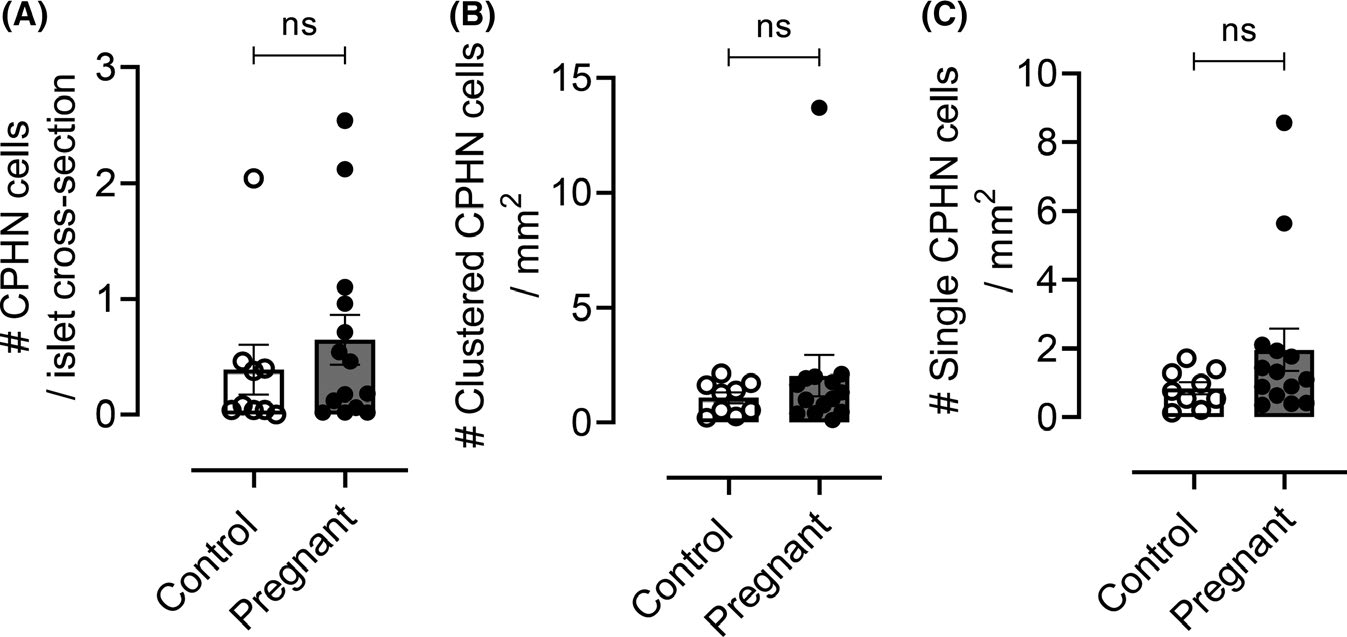
The frequency of CPHN cells in control (non‐pregnant subjects) and pregnant subjects. There was no difference in CPHN cells in any compartment (islets, clusters or as single cells) in pregnant vs. non‐pregnant women (0.6 ± 0.2 vs. 0.4 ± 0.2 CPHN cells/islet section, pregnant vs. non‐pregnant, p = ns (A); 2.1 ± 0.9 vs. 1.0 ± 0.2 clustered CPHN cells/mm^2^, pregnant vs. non‐pregnant, p = ns (B); 2.0 ± 0.6 vs. 0.8 ± 0.2 single CPHN cells/mm^2^, pregnant vs. non‐pregnant, p = ns (C)

Table S3 chromogranin‐positive hormone‐negative (CPHN) cells were detected in both non‐pregnant controls (Figure 2A **)** and pregnant women (Figure 2B). We found no difference in CPHN cells in any compartment (islets, clusters or single cells) of pregnant compared to non‐pregnant women (0.6 ± 0.2 vs. 0.4 ± 0.2 CPHN cells/islet section, pregnant vs. non‐pregnant, p = ns; 2.1 ± 0.9 vs. 1.0 ± 0.2 clustered CPHN cells/mm^2^, pregnant vs. non‐pregnant, p = ns; 2.0 ± 0.6 vs. 0.8 ± 0.2 single CPHN cells/mm^2^, pregnant vs. non‐pregnant, p = ns) (Figure 3A–C).

### 3.3 Effect of gravidity on the frequency of CPHN cells

In our pregnancy cohort, there were 5 women who were gravida 1 (G1) and 3 women who were gravida 2 (G2). In addition, there was 1 gravida 3 (G3), 1 G4, 1 G5 and 1 G14 subject in our cohort. The gravidity of 2 women was not documented. We compared the frequency of CPHN cells in all compartments of pancreas and found no difference in the frequency of CPHN cells between G1 and G ≥ 2 (Figure S3).

## 4 DISCUSSION

Pancreatic non‐hormone expressing endocrine cells (CPHN cells) are found in abundance in normal human pancreas during development.^1^ A resurgence of CPHN cells has been documented in both type 1 diabetes (T1D)^4^ and type 2 diabetes (T2D)^13^ where there is a deficit in the endocrine, specifically the beta cell, complement.^22^, ^23^ As a consequence of these observations, we have previously proposed that CPHN cells are an immature type of endocrine cell representing, in the setting of a pathological beta cell deficiency, an attempted, albeit insufficient, regenerative response, in an effort to restore the beta cell complement.

Pregnancy represents a unique physiological state of beta cell stress. In this study, we report that the frequency of CPHN cells does not change in the setting of human pregnancy uncomplicated by hyperglycaemia, having an overall frequency of ~2.5% CPHN cells (of total endocrine cells) in pregnant women. Our current data also demonstrates that the number of pancreatic endocrine cells per islet section (including pancreatic beta cells as well as endocrine cocktail) remains the same in pregnant vs. non‐pregnant women, suggesting no impact of CPHN cells in contributing to the endocrine/beta cell pool in pregnancy.

In pregnancy, maternal pancreatic β‐cells undergo morphological adaptation to compensate for the excess insulin demand.^19^ Previous studies suggest that rodent and human β cells adapt differently to pregnancy.^12^, ^21^, ^24^, ^25^, ^26^ The mechanisms responsible for this adaptation involve increased replication and decreased apoptosis in rodents,^25^, ^27^ but possibly involve islet neogenesis in humans.^21^ Recently, it has been proposed that ‘altered identity’ of pancreatic β‐cells is another player in the stress‐induced adaptation of pancreatic β‐cells in humans (reviewed in.^28^)

We previously reported that the adaptive increase in β‐cells in human pregnancy is achieved by formation of new islets, which is again confirmed by our current observation that the per cent of clustered β‐cells was increased in pregnancy. Interestingly, our current data also indicated a decreased proportion of endocrine cocktail cells in pregnancy, suggesting that adaptive changes of endocrine pancreas might drive transdifferentiation of other endocrine cells to β‐cells to cope with the excess insulin demand of human pregnancy. Therefore, it is possible that the CPHN cells present in pregnancy may be indicative of ‘endocrine cells in transition’ with altered identity rather than new cell formation as was observed in humans with T1D and T2D.^3^, ^4^

Here we report that the frequency of CPHN cells in pancreas of pregnant women is not increased in comparison with age‐matched non‐pregnant control women. One possible explanation for the lack of an increase in CPHN cells in pregnant women might be the ‘gestational window’ of the selected cases in our study. The average gestational age of the pregnant women in our study was 24.5 ± 2.7 weeks (ranging from 10 to 40 weeks). Early human pregnancy (the first two trimesters) is an anabolic phase, during when pancreatic β‐cell activity increases; as such, an enhanced insulinotropic effect of glucose is seen during this phase, whereas insulin sensitivity is either not altered or enhanced.^29^ In contrast, the decrease in insulin sensitivity that occurs in later gestation (known as the catabolic phase) may enhance the workload of β‐cells to compensate for the increased insulin demand.^29^ A recent report has described, in the setting of pregnancy‐induced insulin resistance in the liver‐specific insulin receptor knock‐out LIRKO mouse, increased ductal epithelial‐derived clustered small islet clusters, suggesting that the ductal compartment contributes to bolster beta cell mass through neogenesis during pregnancy.^30^ In human pregnancy, we might anticipate an increase in CPHN cells either in the period of very early gestation (5–10 weeks) or in the most advanced gestational period (35–40 weeks), as it is expected that β‐cell neogenesis or stress‐induced dedifferentiation would also peak at those stages. Our study, however, included only a few cases that corresponded to those specific gestational windows. Therefore, the insufficient number of appropriate cases may have resulted in a failure to detect a surge in CPHN cells during those critical time points.

In conclusion, our study found no increase in the frequency of CPHN cells in pancreas of women in response to the increased metabolic workload of pregnancy when compared with non‐pregnant control women. Our finding suggests that this potential beta cell regenerative mechanism is not that by which the increased beta cell mass of pregnancy is achieved, although further studies utilizing pancreas from pregnant women during the critical early and late gestational windows are warranted. Interestingly, however, an increase in the percentage of clustered beta cells was found in pregnancy, with a decrease in frequency of other endocrine cells in clusters, suggesting that a compensatory shift occurs to bolster the beta cell component in order to compensate for the increased insulin demand of pregnancy.

## CONFLICT OF INTEREST

The authors report no conflicts of interest in this work.

## AUTHOR CONTRIBUTION

ASMM and KZ performed the studies analysed the data and wrote the first draft of the manuscript. RAR procured the autopsy pancreas samples and edited the manuscript. SD and AEB contributed to study design, data interpretation and the writing of the manuscript. All authors reviewed and approved the final version of the manuscript. Alexandra E Butler is the guarantor of this work.

## ETHICAL APPROVAL

Institutional Review Board (IRB) approval from the Mayo Clinic and University of California Los Angeles (UCLA) was obtained. Both the Mayo Clinic IRB and the UCLA IRB waived the requirement for written informed consent for this study due to the following reasons: (1) According to the FDA definition of human subjects, a human subject means a living individual and therefore (2) research on human autopsy subjects is considered by the IRB to be ‘exempt’ as defined by federal regulation 45 CFR 46, as autopsy subjects do not meet the FDA definition of human subjects.

## CONSENT TO PARTICIPATE

Not applicable.

## CONSENT FOR PUBLICATION

All authors gave their consent to publish this work.

## CODE AVAILABILITY

Not applicable.

## Supporting information

Fig S1

Fig S2

Fig S3

Tab S1

Tab S2

Tab S3

## Data Availability

All relevant data are within the manuscript and its Supporting Information files.
