# Supplementary figures and images for: Chromogranin A‐positive hormone‐negative endocrine cells in pancreas in human pregnancy
Source: Endocrinol Diabetes Metab. 2021 Jan 6;4(2):e00223. doi: 10.1002/edm2.223 (PMC8029563; doi:10.1002/edm2.223)

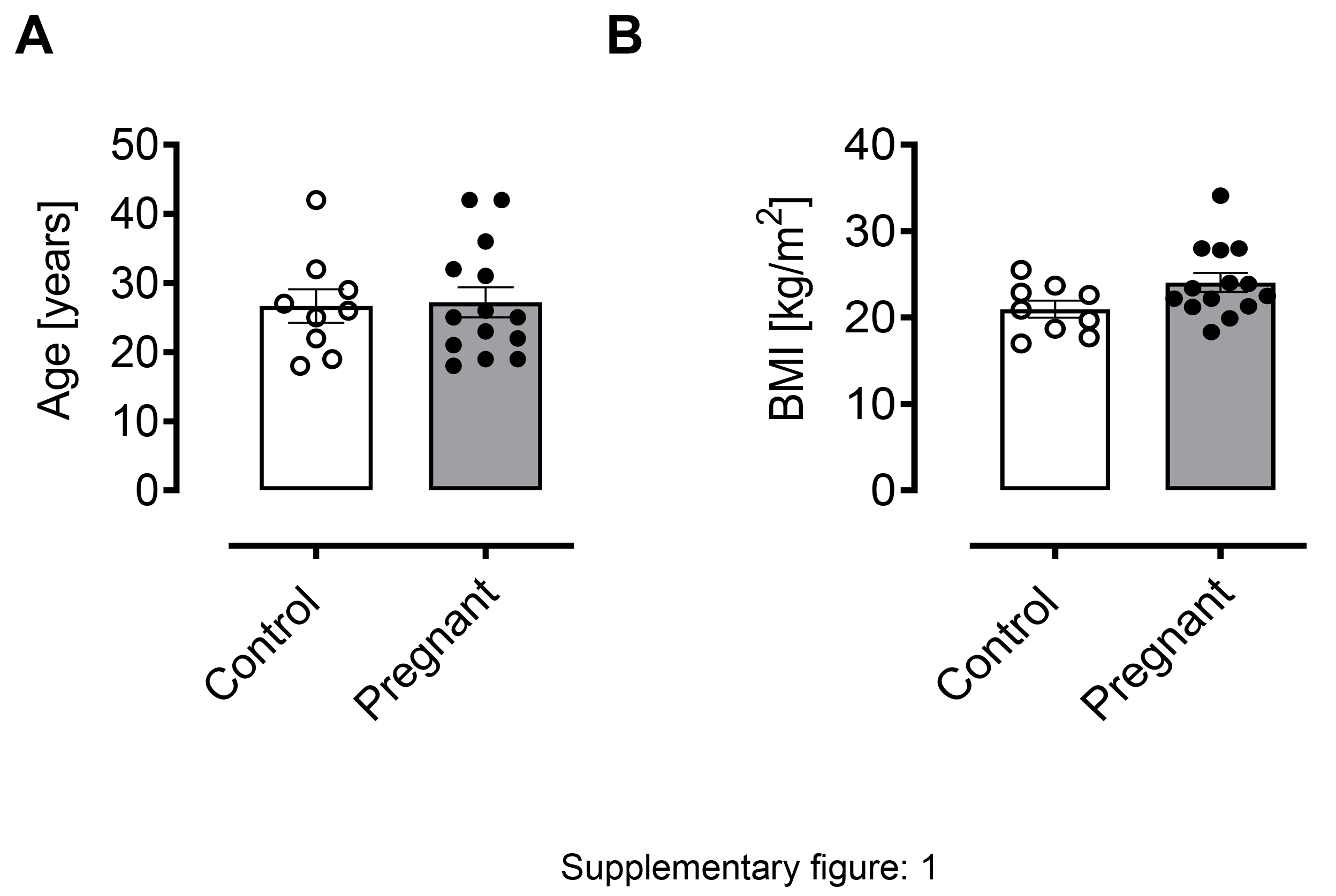

Supplement: Supplementary file 1 — Fig S1 [file EDM2-4-e00223-s003.tif]

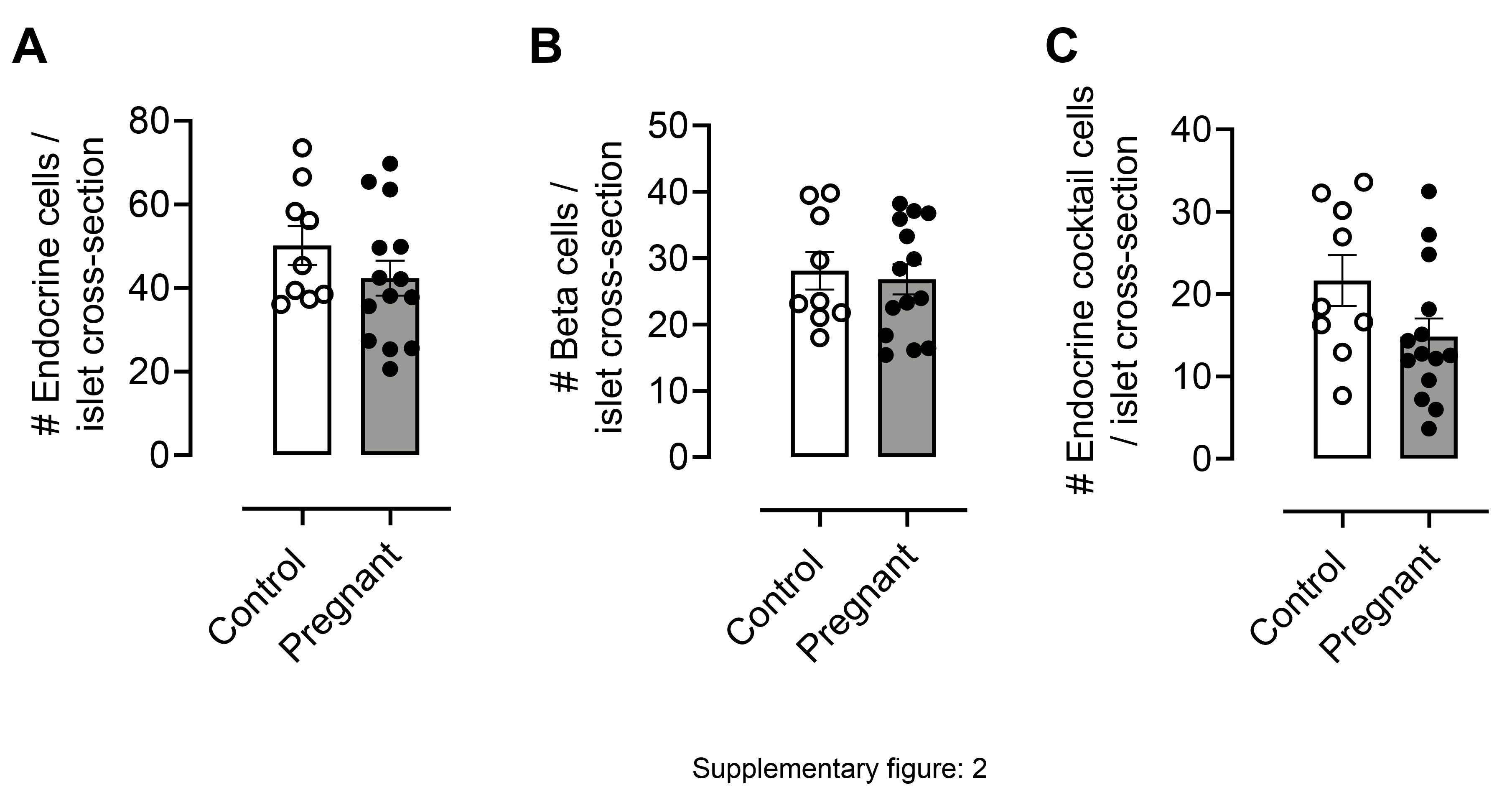

Supplement: Supplementary file 2 — Fig S2 [file EDM2-4-e00223-s005.tif]

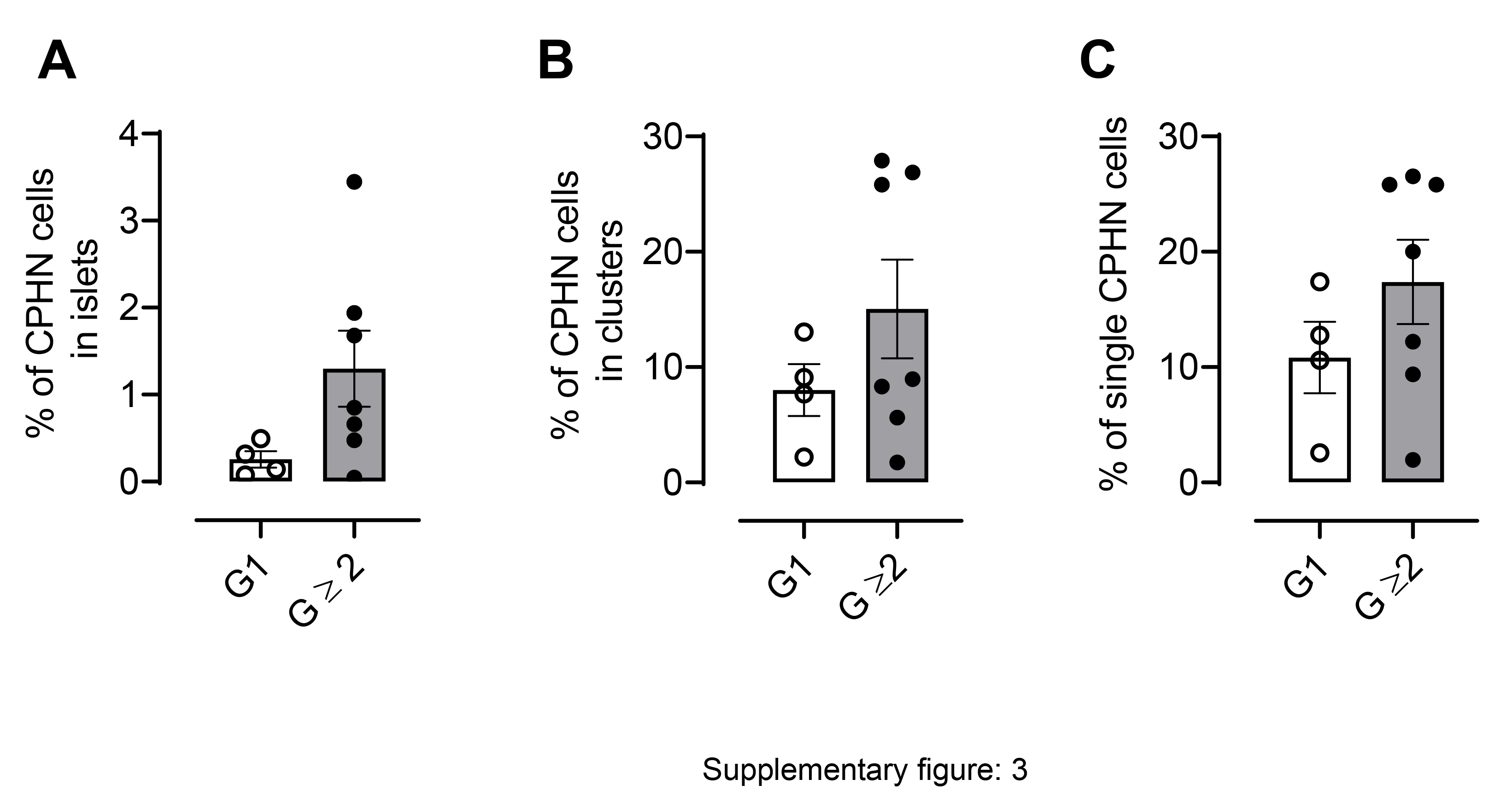

Supplement: Supplementary file 3 — Fig S3 [file EDM2-4-e00223-s002.tiff]
