# Supplementary material for: Chromogranin A‐positive hormone‐negative endocrine cells in pancreas in human pregnancy
Source: Endocrinol Diabetes Metab. 2021 Jan 6;4(2):e00223. doi: 10.1002/edm2.223 (PMC8029563; doi:10.1002/edm2.223)
Supplement: Supplementary file 4 — Tab S1 [file EDM2-4-e00223-s006.docx]

**Supplementary Table 1:** List of primary and secondary antibodies used in this study.

| Primary antibodies | | | | | |
| --- | --- | --- | --- | --- | --- |
| **Name** | **Host** | **Dilution** | **Company** | **Catalogue** | **Research Resource Identifier (RRID)** |
| Insulin | Guinea pig | 1:200 | Abcam, Cambridge, MA | 7842 | AB_306130 |
| Glucagon | Mouse | 1:1000 | Sigma-Aldrich, St. Louis, MO | G2654-0.2ML | AB_259852 |
| Somatostatin | Rat | 1:300 | EMD Millipore, Billerica, MA | MAB354 | AB_2255365 |
| Pancreatic polypeptide | Goat | 1:3000 | Everest Biotech, Ramona, CA | EB06805 | AB_2169058 |
| Ghrelin | Rat | 1:50 | R&D Systems, Minneapolis, MN | MAB8200 | AB_2637039 |
| Chromogranin A | Rabbit | 1:200 | Novus Biologicals, Littleton, CO | NB120-15160 | AB_789299 |
| **Secondary antibodies (Jackson Immuno- Research, West grove, PA)** | | | | | |
| **Name** | **Host** | **Dilution** | **Catalogue** | **To detect** | **Research Resource Identifier (RRID)** |
| Cy3 | Rabbit | 1:200 | 711-166-152 | chromogranin A | AB_2313568 |
| Fluorescein isothiocyanate (FITC) | Mouse | 1:200 | 715-096-150 | Glucagon | AB_2340795 |
|  | Rat |  | 712-096-150 | Somatostatin | AB_2340653 |
|  | Goat |  | 705-096-147 | Pancreatic polypeptide | AB_2340402 |
|  | Rat |  | 712-096-150 | Ghrelin | AB_2340653 |
| Cy5 | Guinea Pig | 1:100 | 706-606-148 | Insulin | AB_2340477 |
