# Supplementary material for: Chromogranin A‐positive hormone‐negative endocrine cells in pancreas in human pregnancy
Source: Endocrinol Diabetes Metab. 2021 Jan 6;4(2):e00223. doi: 10.1002/edm2.223 (PMC8029563; doi:10.1002/edm2.223)
Supplement: Supplementary file 5 — Tab S2 [file EDM2-4-e00223-s001.docx]

**Supplementary Table 2:** The comparison of endocrine cells in islets and clustered in non-pregnant and pregnant subjects.

|  | Non-pregnant | Pregnant |
| --- | --- | --- |
| %Total clustered endocrine cells  of total endocrine cells in all compartment | 3.5 ± 0.5 | 3.8 ± 0.7 |
| % of clustered β cells  of total clustered endocrine cells | 31.8 ± 5.0 | 46.6 ± 5.0* |
| % of clustered endo-cocktail cells  of total clustered endocrine cells | 36.2 ± 4.02 | 57.02 ± 6.8** |
| Total Endocrine Cells /Islet Cross-section | 50.1 ± 4.4 | 42.3 ± 4.1 |
| Beta cells/ Islet Cross-section | 28.1 ± 2.7 | 26.8 ± 2.2 |
| Endocrine Cocktail cells/ Islet Cross-section | 21.6 ± 3.1 | 14.8 ± 2.2 |

*, p < 0.05; **, p < 0.01
