# Supplementary material for: Chromogranin A‐positive hormone‐negative endocrine cells in pancreas in human pregnancy
Source: Endocrinol Diabetes Metab. 2021 Jan 6;4(2):e00223. doi: 10.1002/edm2.223 (PMC8029563; doi:10.1002/edm2.223)
Supplement: Supplementary file 6 — Tab S3 [file EDM2-4-e00223-s004.docx]

**Supplementary Table 3**: The composition of CPHN cells in islets and clustered cells in non-pregnant and pregnant subjects.

|  | CPHN cells  / islet Cross-section | Cluster CPHN  cells / mm2 | Single CPHN  cells / mm2 |
| --- | --- | --- | --- |
| Pregnant subjects | 0.6 ± 0.2 | 2.1 ± 0.9 | 2.0 ± 0.6 |
| Non-pregnant Subjects | 0.4 ± 0.2 | 1.0 ± 0.2 | 0.8 ± 0.2 |
